# Case Report: Severe psoriasis skin lesions following ofatumumab treatment for multiple sclerosis

**DOI:** 10.3389/fimmu.2025.1595947

**Published:** 2025-08-12

**Authors:** Wioletta Katarzyna Żukowicz-Wyrwa, Karolina Markiet, Tatsiana Damps, Aleksandra Ciarka, Bartosz Karaszewski, Roman Nowicki

**Affiliations:** ^1^ Department of Adult Neurology, Faculty of Medicine, Medical University of Gdansk, Gdansk, Poland; ^2^ Department of Adult Neurology, University Clinical Center in Gdansk, Gdansk, Poland; ^3^ 2^nd^Department of Radiology, Medical University of Gdansk, Gdansk, Poland; ^4^ Division of Radiology, University Clinical Center in Gdansk, Gdansk, Poland; ^5^ Department of Dermatology, Venereology and Allergology, Faculty of Medicine, Medical University of Gdansk, Gdansk, Poland; ^6^ Department of Dermatology, Venereology and Allergology, University Clinical Center in Gdansk, Gdansk, Poland; ^7^ Department of Pathomorphology, Faculty of Medicine, Medical University of Gdansk, Gdansk, Poland

**Keywords:** multiple sclerosis, ofatumumab, psoriais, anti-CD 20 antibodies, adverse effect

## Abstract

The article explores a case study of a patient diagnosed with relapsing-remitting multiple sclerosis (RRMS) who, while receiving treatment with the anti-CD20 monoclonal antibody ofatumumab, developed psoriatic skin lesions. Initially, sharply demarcated, scaly, erythematous lesions were observed only in the anogenital area. After several weeks, additional lesions appeared on the ear lobes, scalp, and lower legs following subsequent administrations of ofatumumab. A comprehensive differential diagnosis of the skin lesions was performed, which included consideration of autoimmune chronic diseases, infectious disease manifestations, and skin carcinomas. Biopsies of the lesions were obtained and underwent histopathological examination. Based on the clinical presentation and further investigations, it was concluded that the skin lesions were an atypical adverse reaction to ofatumumab, manifesting as psoriasis. In addition to presenting this case, we analyze the spectrum of common adverse reactions associated with ofatumumab, as well as the occurrence of psoriasis following other anti-CD20 therapies. To the best of our knowledge, this is a rare, documented instance of psoriasis occurring in a patient receiving ofatumumab for the treatment of multiple sclerosis.

## Introduction

Recent advancements in multiple sclerosis treatment involve anti-CD20 monoclonal antibodies targeting CD20+ B and T cells to reduce inflammation. Psoriasis cases have been observed with rituximab or ocrelizumab. Ofatumumab’s safety, efficacy, and tolerability were evaluated in the ASCLEPIOS I and II trials (1). However, its full adverse effect profile remains unclear due to its relatively recent introduction. The patient provided consent for publication.

## Case description

The 35-year-old patient, diagnosed with RRMS at 17, was admitted to the Department of Neurology in September 2022 due to weakness in the left limbs that began a few days before admission. The patient had been managed with interferon beta therapy since disease onset and, after 15 years, was switched to dimethyl fumarate due to a relapse of MS and poor treatment tolerance, including flu-like symptoms and depressive episodes. Neurological examination revealed mild left-sided limb weakness and persistent right lower limb weakness. Additionally, the patient presented with ataxia in the upper limbs and impaired proprioceptive sensation in all limbs. The Expanded Disability Status Scale (EDSS) score was 3.0. Methylprednisolone was administered intravenously at 1 g for 5 days, leading to slight improvement in left limb weakness.

**Figure 1:**
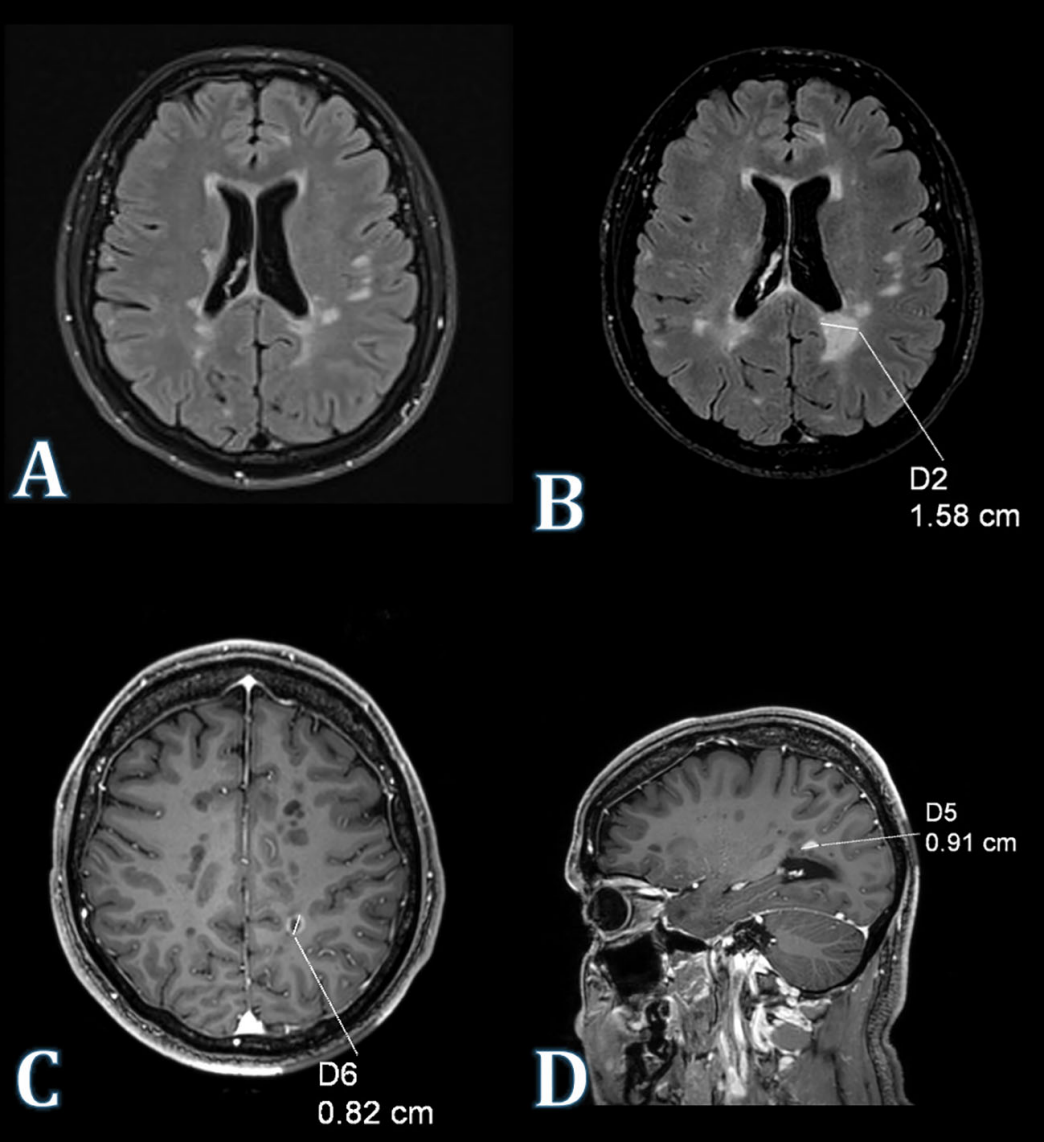
MRI of the brain. **(A)** Axial FLAIR image (2021) depicts a demyelinating lesion located proximal to the posterior horn of the left lateral ventricle. **(B)** Axial FLAIR image acquired in 2022 illustrates an enlargement of the demyelinating lesion situated in the vicinity of the posterior horn of the left lateral ventricle. **(C)** Axial contrast-enhanced T1WI image displays peripheral enhancement of the demyelinating lesion following intravenous administration of a gadolinium-based contrast agent. **(D)** Sagittal contrast-enhanced T1WI image of the same lesion.

A follow-up contrast-enhanced brain MRI, performed on March 7, 2022, revealed progression of demyelinating lesions in the left hemisphere. Compared to a 2021 MRI, an enlargement of two demyelinating lesions in the temporal and posterior horn of the left lateral ventricle was noted. Peripheral enhancement of the lesions was observed after intravenous contrast (**Figure 1**).

Due to disease progression and ongoing clinical and radiological activity, the treatment regimen was modified. Dimethyl fumarate was discontinued, and ofatumumab was initiated on January 16, 2023. After the first dose, flu-like symptoms were reported, but subsequent administrations were well tolerated with no adverse effects.

**Figure 2:**
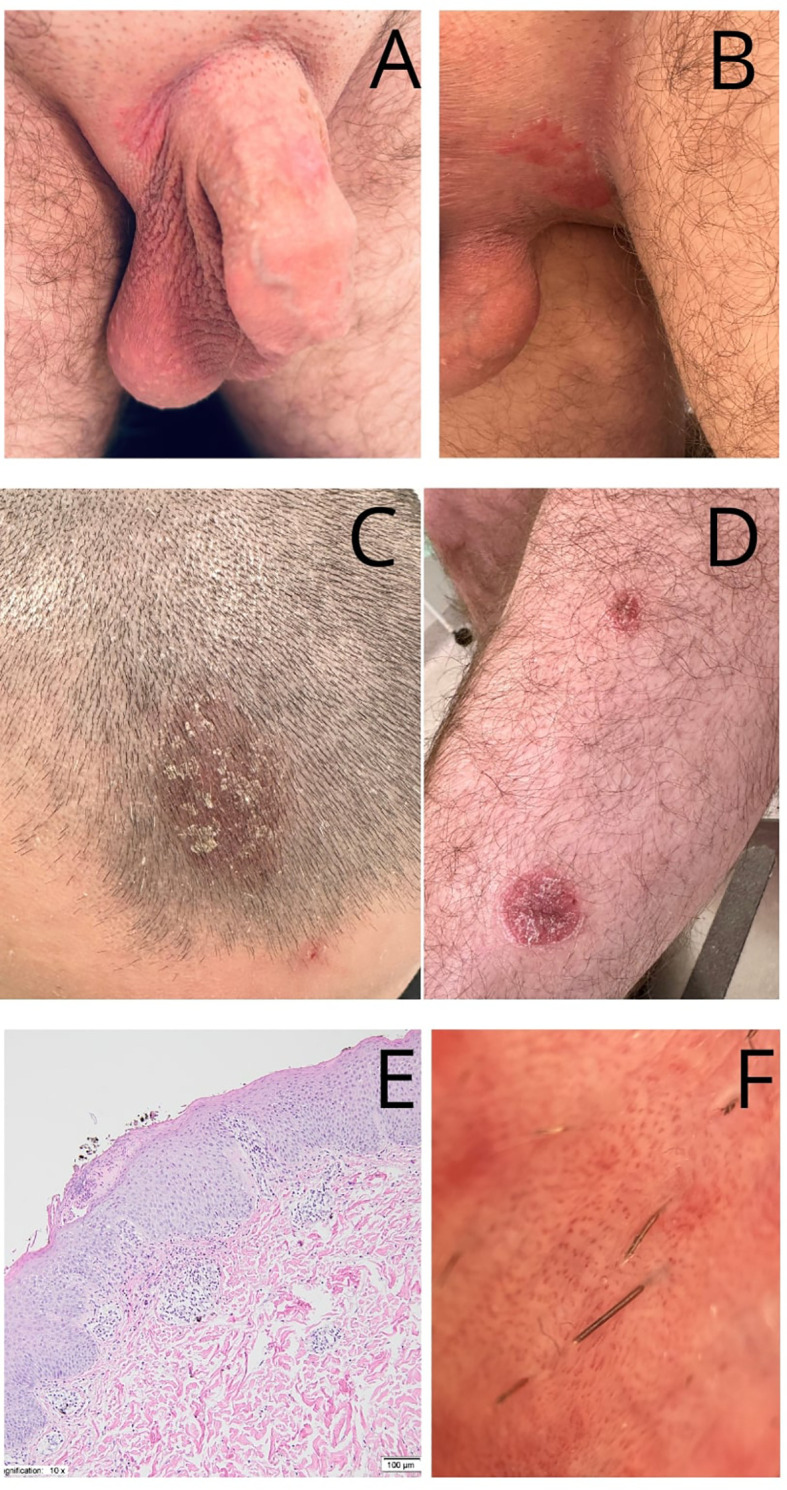
Diagnostic and clinical findings. **(A, B)** Psoriatic skin lesions - well-demarcated, bright red, thin plaques of the external genitalia. **(C)** red, thickened, well-demarcated plaque with overlying white-yellowish scales, affecting frontal part of the scalp. **(D)** well-demarcated, bright red, thin plaques of the left lower leg. **(E)** Histopathological examination revealed an acanthotic epidermal hyperplasia accompanied by hyperkeratosis and parakeratosis, elongation of the rete ridges and thinning of the suprapapillary plates. Additionally, perivascular lymphocytic infiltrates are observed in the upper and mid-dermis. The papillary dermis contains dilated and tortuous blood vessels (H&E staining, magnification 10x). **(F)** Dermoscopic manifestations of genital psoriatic skin lesions: regular arrangement of red-dotted/bulbed vessels; light-red background. Figures were examined by Heine DELTAone digital dermoscope (Heine, Germany) with a magnification of ×10.

After approximately five months of treatment, the patient developed pruritic, burning skin lesions in the perianal, genital, and groin areas, which later spread to the scalp, retroauricular regions, and lower legs. Treatment with ofatumumab was immediately discontinued upon the appearance of skin lesions. Discontinuation of ofatumumab, along with symptomatic treatment, led to gradual improvement of the lesions (**Figures 2A–D**).

A comprehensive differential diagnosis was conducted. The patient consulted dermatology, venereology, and allergology specialists. Nails, mucosa, and joints were normal. Two viral warts were observed on the right hand, but no further symptoms were noted. Routine laboratory investigations showed no significant abnormalities, including no eosinophilia. Serological tests for hepatitis A, B, and C viruses, HIV, and Chlamydia trachomatis were all negative. A mycological test from the groin was negative as well.

Histopathological examination revealed acanthotic epidermal hyperplasia with hyperkeratosis and parakeratosis, elongation of the rete ridges, and thinning of the suprapapillary plates (**Figure 2E**). Perivascular lymphocytic infiltrates were present in the upper and mid-dermis, and the papillary dermis contained dilated, tortuous blood vessels. Dermoscopic examination of the genital lesions revealed a regular arrangement of red-dotted vessels on a light-red background, consistent with psoriasis (**Figure 2F**).

Ofatumumab was discontinued, and the medication was withdrawn for several weeks, during which partial improvement in the skin lesions was observed. Initially, other potential etiologies for the lesions were considered, leading to the decision to reintroduce ofatumumab after several weeks of observation and a reduction in the skin lesions. Unfortunately, upon reinitiating ofatumumab, a recurrence of the skin lesions was observed. Ultimately, after approximately two months, a diagnosis of psoriasis was confirmed. Topical corticosteroids, antifungal agents, salicylic acid, and emollients were administered with good clinical response. However, complete remission of the skin lesions was not achieved. As a result, a decision was made to switch treatment to ozanimod (a sphingosine 1-phosphate receptor modulator). The patient remains under regular follow-up at the dermatology outpatient clinic.

## Discussion

Ofatumumab is generally considered safe and well tolerated, with mild adverse effects. Among the most common adverse reactions, occurring in at least 10% of patients, are injection-related reactions (IRRs) and upper respiratory tract infections, particularly with the initial dose (2, 3). Other commonly reported adverse events include nasopharyngitis, upper respiratory tract infections, urinary tract infections, and COVID-19 (2, 4). The group of anti-CD20 antibodies is not homogeneous. Individual antibodies differ not only in structure and in their CD20 antigen binding sites but also in the mechanisms leading to B-cell depletion.

Ocrelizumab is a humanized antibody that primarily works through antibody-dependent cellular cytotoxicity (ADCC). Ofatumumab, on the other hand, is a fully human antibody and acts mainly through complement-dependent cytotoxicity (CDC). Notably, there have been no reports of opportunistic infections, venereal diseases, or hepatitis B reactivation with ofatumumab, unlike ocrelizumab, another anti-CD20 therapy Kliknij lub naciśnij tutaj, aby wprowadzić tekst (4, 5) Kliknij lub naciśnij tutaj, aby wprowadzić tekst.

It is well-recognized that the use of biological antibodies, regardless of the clinical indication, increases the incidence of adverse drug reactions, which may manifest as skin symptoms ranging from localized erythema to more systemic reactions (6). Based on clinical observations, some patients treated with ofatumumab may experience transient, self-limiting cutaneous changes, primarily localized to the injection site. Moreover, the literature indicates that treatment with B-cell-depleting agents such as ocrelizumab and rituximab is associated with an increased risk of psoriasis (6, 7). According to Herzum et al., approximately 20 cases of rituximab-induced psoriasis have been reported, with the onset occurring between 2 weeks and 2 years following treatment initiation (8). Additionally, Kölsche et al. described a case of a patient who developed psoriasis following treatment with ofatumumab, with symptoms improving concurrently with the normalization of CD19+ B cell levels (9).

The authors suggest that this observation may indicate a potential role of regulatory B cells in the pathogenesis of the disease. The depletion of regulatory B cells, which are crucial for immune regulation and skin protection, may contribute to psoriasis by reducing their suppressive effects on inflammation and enhancing the activation of other immune cells, such as T cells. This phenomenon could trigger or exacerbate psoriasis, leading to more pronounced skin symptoms and increased disease severity (6). Notably, reports indicate that B-cell repopulation occurs earlier with ofatumumab compared to other B-cell-depleting therapies (1).

Pfeuffer et al. have suggested a potential immunological mechanism underlying the development of psoriasis following treatment with ocrelizumab, another anti-CD20 monoclonal antibody (10). Although both ocrelizumab and ofatumumab target CD20 receptors, their mechanisms of action differ, as described earlier. According to Pfeuffer, psoriasis is characterized by strong activation of interleukin (IL)-12 family cytokines and tumor necrosis factor-alpha (TNF-α) and therefore responds well to therapies targeting these pathways (10). Conversely, MS derives only modest benefit from IL-23 blockade and may even deteriorate with TNF-α inhibition (10). Importantly, regulatory B cells have been shown to suppress IL-23 signaling in psoriasis (10). Through this mechanism, unfavorable alterations in the immune network beyond the B-cell compartment may occur, promoting the development of secondary autoimmunity in genetically or immunologically susceptible individuals. It is possible that the pathomechanism of psoriasis in patients receiving ofatumumab mirrors that associated with ocrelizumab.

In one of the previously reported cases, treatment was switched from dimethyl fumarate to ofatumumab; however, psoriasis developed within one week of the first dose (9). In another case, the authors reported a woman who developed psoriasis three months after the initiation of ofatumumab therapy (8). In contrast, our patient developed psoriatic lesions after five months of treatment. Improvement in skin lesions was observed following discontinuation of ofatumumab. It is worth noting that in another reported case of psoriasis following the first full dose of ocrelizumab, the authors also decided to discontinue further administration of the drug (11). However, in their discussion, they emphasized that there is no solid evidence to definitively answer the question of whether ocrelizumab therapy should be continued or discontinued (11). Similar to Kölsche, the authors also highlighted the potential role of anti-IL-17 antibody (secukinumab), which is approved for the treatment of psoriasis (9, 11). Although further research is needed, there is some evidence suggesting that secukinumab may be beneficial for patients with multiple sclerosis (9, 11). We believe that further observation and research are warranted to assess the risk of psoriasis associated with ofatumumab and other anti-CD20 antibodies, as well as to establish clear guidelines for the management and continued treatment of multiple sclerosis in such cases.

## Conclusion

In this case, based on the clinical presentation and histopathological findings, the skin changes were diagnosed as drug-induced psoriasis caused by ofatumumab. Particular attention should be given to the sudden onset of extensive skin lesions initially localized to the anogenital region. A thorough differential diagnosis was conducted, excluding infections, sexually transmitted diseases, and malignancies.

To the best of our knowledge, this is the first documented case of acute skin changes confined to the anogenital region and presenting as psoriasis, as a manifestation of adverse effects related to ofatumumab. Furthermore, this represents one of the few documented cases of newly onset psoriasis in a patient undergoing ofatumumab therapy for multiple sclerosis.

## Data Availability

The datasets presented in this article are not readily available because of ethical and privacy restrictions. Requests to access the datasets should be directed to the corresponding author/s.
